# β‐Elemene Triggers Drp1‐Dependent Mitochondrial Fission Through CDK1/Cyclin B1 Signaling in Cervical Cancer Cells

**DOI:** 10.1002/jbt.71032

**Published:** 2026-07-27

**Authors:** Zhifang Li, Ling Tang, Mingyan Wang, Qiong Yu, Chen Chen, Jiyi Xia, Xiaolan Yu

**Affiliations:** ^1^ Department of Obstetrics and Gynecology, The Affiliated Traditional Chinese Medicine Hospital Southwest Medical University Luzhou City Sichuan Province China; ^2^ School of Traditional Chinese Medicine Dazhou Vocational College of Chinese Medicine Dazhou City Sichuan Province China; ^3^ The Affiliated Traditional Chinese Medicine Hospital Southwest Medical University Luzhou City Sichuan Province China; ^4^ Department of Technology and Social Service Dazhou Vocational College of Chinese Medicine Dazhou City Sichuan Province China; ^5^ Dazhou Chinese Medicine Research and Development Center Dazhou City Sichuan Province China

**Keywords:** CDK1/Cyclin B1, cervical cancer, Drp1, mitochondrial fission, β‐elemene

## Abstract

Mitochondrial dynamics, regulated by fission and fusion, are frequently altered in cancers, influencing cell survival and metabolism. The sesquiterpene β‐elemene exhibits anti‐tumor activity, but its effect on mitochondrial dynamics in cervical cancer is unknown. This study investigated whether β‐elemene exerts its anti‐tumor effects by disrupting mitochondrial homeostasis. We found that β‐elemene treatment dose‐dependently reduced viability and increased lactate dehydrogenase release in HT‐3 and Caski cervical cancer cells. In HT‐3 cells, β‐elemene induced mitochondrial oxidative stress, impaired respiratory function, and triggered extensive mitochondrial fragmentation. Mechanistically, β‐elemene promoted phosphorylation of dynamin‐related protein 1 (Drp1) at Ser616 and its translocation to mitochondria. Furthermore, β‐elemene enhanced the interaction between cyclin‐dependent kinase 1 (CDK1) and cyclin B1. Genetic silencing of CDK1 abrogated β‐elemene‐induced Drp1 activation, mitochondrial fragmentation, and bioenergetic deficits. Collectively, these data identify a novel pathway through which β‐elemene drives CDK1‐dependent Drp1 activation, leading to excessive mitochondrial fission and dysfunction in cervical cancer cells.

## Graphical abstract

This study reveals a novel molecular mechanism by which the natural sesquiterpene β‐Elemene exerts anti‐tumor effects in cervical cancer. β‐Elemene treatment promotes the activation of the CDK1/Cyclin B1 kinase complex. Activated CDK1/Cyclin B1, in turn, phosphorylates the mitochondrial fission protein Drp1 at serine 616. Phosphorylated Drp1 translocates to mitochondria, triggering excessive mitochondrial fission. This leads to severe mitochondrial fragmentation, bioenergetic failure (including ATP depletion), and ultimately, cervical cancer cell death. Our findings identify the CDK1‐Drp1‐mitochondrial fission axis as a critical signaling pathway and a potential therapeutic target in cervical cancer.

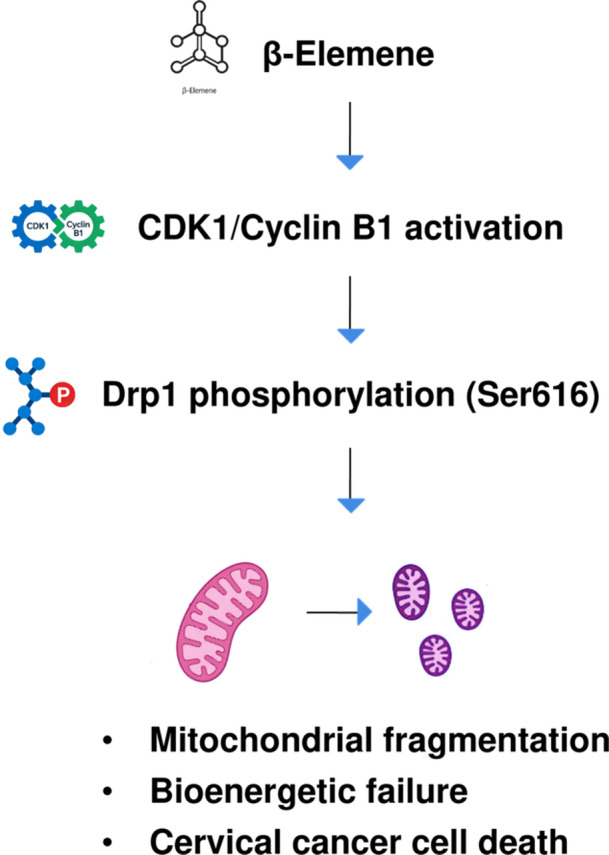

## 1 Introduction

Mitochondria are far more than the simple power plants of the cell. These highly dynamic organelles undergo continuous cycles of fission and fusion, processes collectively termed mitochondrial dynamics, which are fundamental to maintaining cellular health, energy metabolism, and programmed cell death [1]. The precise balance between these opposing processes governs mitochondrial morphology, distribution, and function [1]. Dynamin‐related protein 1 (Drp1) serves as the master regulator of mitochondrial fission [2]. In its inactive cytosolic state, Drp1 is recruited to the mitochondrial outer membrane upon specific phosphorylation events, notably at serine 616 (Ser616), where it oligomerizes to constrict and divide mitochondria [3]. Excessive or unopposed Drp1‐mediated fission leads to mitochondrial fragmentation, a phenomenon intimately linked to bioenergetic crisis, the release of pro‐apoptotic factors, and ultimately, cellular demise [4].

Mitochondrial dynamics are significantly altered in cancer to facilitate the malignant phenotype [5, 6]. Tumors often display a transition to fragmented mitochondrial networks, which are associated with elevated glycolysis, heightened reactive oxygen species (ROS) generation, and resistance to apoptosis [5, 6]. This disjointed condition is believed to provide a metabolic advantage under stress and aid in the dispersion of mitochondria during fast cellular division. Cervical cancer, a major source of cancer‐related death among women worldwide, exemplifies this metabolic reprogramming [7]. Although the involvement of classical signaling pathways in cervical carcinogenesis is well‐established, the impact of dysregulated mitochondrial dynamics, especially via Drp1, is a significant but under‐investigated area. Recent research indicates that addressing these processes may provide a new therapeutic vulnerability [8].

Numerous natural chemicals are being considered as potential anti‐cancer medicines owing to their capacity to specifically target cancer cell metabolism and trigger mitochondrial‐mediated apoptosis [9]. β‐Elemene, a sesquiterpene derived from the traditional Chinese medicinal plant Curcuma wenyujin, is one such chemical [10]. It has shown extensive anti‐tumor efficacy against several malignancies, including lung, glioma, and breast cancers, by the inhibition of proliferation, induction of cell cycle arrest, and facilitation of apoptosis [11, 12]. Several studies indicate the involvement of mitochondria in the action of β‐elemene. It has been shown to impair mitochondrial membrane potential and initiate the intrinsic apoptotic pathway [13]. The exact biochemical mechanism via which β‐elemene affects the fundamental processes of mitochondrial dynamics—particularly the fission and fusion machinery—in any disease, including cervical cancer, remains completely unexplained. The issue of whether its cytotoxic effects result from directly interfering with proteins such as Drp1 remains unresolved.

The activation of Drp1 is controlled by complex post‐translational modifications, with phosphorylation being a central regulatory mechanism. Cyclin‐dependent kinase 1 (CDK1), in complex with its regulatory partner cyclin B1, is a key mitotic kinase traditionally known for governing cell cycle progression from G2 to M phase [14]. Intriguingly, a novel, non‐canonical role for CDK1 has surfaced in recent years. Beyond the nucleus, active CDK1/cyclin B1 can phosphorylate mitochondrial substrates during mitotic arrest and cellular stress, influencing apoptosis and organellar integrity [15]. Critically, emerging evidence from non‐cancer systems indicates that CDK1 can directly phosphorylate Drp1 at Ser616, driving mitochondrial fission in models of neuronal injury and cardiomyocyte apoptosis [16, 17]. This intersection of cell cycle machinery and mitochondrial shape regulation represents a compelling signaling axis. Whether this CDK1/Drp1 pathway is operational in cancer cells and can be pharmacologically targeted by agents like β‐elemene is a tantalizing possibility that has not been investigated. This gap in knowledge is particularly relevant for cervical cancer, where the expression and activity of CDK1/cyclin B1 are frequently dysregulated [18].

Based on the known anti‐cancer effects of β‐elemene, the dysregulation of mitochondrial dynamics in cancer, and the role of CDK1 in regulating Drp1, we propose that β‐elemene affects cervical cancer cells by hijacking the CDK1/cyclin B1 signaling module, causing pathological Drp1‐mediated mitochondrial fission and irreversible organellar dysfunction and cell death. This research aims to investigate the relationship between β‐elemene and mitochondrial dynamics in cervical cancer, a previously unreported link. We identify a linear signaling route beyond correlated evidence. Testing the idea that β‐elemene's anti‐tumor effect is mediated by the CDK1/cyclin B1‐Drp1‐mitochondrial fission axis is unique. First, we measured baseline Drp1 activity in clinical cervical cancer samples. The cytotoxic and mitochondrial effects of β‐elemene were extensively assessed in cervical cancer cell lines. We used genetic loss‐of‐function tests to show that CDK1 is crucial for transducing β‐elemene's signal to Drp1, causing mitochondrial fragmentation and bioenergetic collapse. This study reveals a potential natural compound's mechanism of action and identifies the CDK1‐Drp1 interface as a possible cervical cancer treatment target.

## 2 Materials and Methods

### 2.1 Cellular Cultivation and Reagents

The human cervical cancer cell lines HT‐3 and Caski were obtained from the American Type Culture Collection (ATCC). HeLa cells (ATCC CCL‐2) were cultured in DMEM (Gibco, 11965092). HT‐3 cells were sustained in McCoy's 5 A medium, while Caski cells were cultivated in RPMI‐1640 medium. All growth medium were augmented with 10% FBS (Gibco, 10270106) and 1% penicillin‐streptomycin solution. Cells were maintained at 37°C in a humidified incubator with a 5% CO_2_ environment and regularly assessed for mycoplasma contamination. β‐elemene (purity > 98%, MedChemExpress, HY‐N0102), a sesquiterpene, was solubilized in dimethyl sulfoxide (DMSO) to provide a 200 mM stock solution, which was preserved at −20°C and diluted in new culture medium before application. The ultimate concentration of DMSO in all treatments remained below 0.1% (v/v), a level that had no discernible impact on cell viability in control studies.

### 2.2 Clinical Tissue Specimens

A cohort of eight paired cervical squamous cell carcinoma specimens and corresponding adjacent non‐tumor mucosal tissues was obtained from the hospital's tissue biobank with informed patient permission and Institutional Review Board clearance. All samples were obtained during therapeutic surgical resections, rapidly frozen in liquid nitrogen, and preserved at ‐80°C for protein analysis, or fixed in 10% neutral‐buffered formalin and embedded in paraffin for immunohistochemical examination.

### 2.3 Cell Viability and Cytotoxicity Assays

For viability evaluations, HT‐3, Caski, and HeLa cells were inoculated onto 96‐well plates at a density of 5 × 10^3^ cells per well. After a 24‐h attachment period, cells were subjected to a gradient of β‐elemene concentrations (0, 20, 40, 60, 80 µM) for 48 h. Cell viability was later assessed with a Cell Counting Kit‐8 (CCK‐8, Dojindo, CK04). Ten microlitres of the CCK‐8 reagent were added to each well, and the plates were incubated for 2 h at 37°C. Absorbance was measured at 450 nm using a microplate reader (BioTek). Cytotoxicity was assessed concurrently by quantifying the activity of lactate dehydrogenase (LDH) released into the culture supernatant. The medium was obtained after a 48‐h treatment, and LDH activity was measured using a commercial kit (Roche, 11644793001) in accordance with the manufacturer's guidelines. The resultant data were normalized to the total cellular protein level, ascertained by a BCA test (Pierce, 23225), and are presented as units per milligram of protein (U/mg protein).

### 2.4 Quantitative Real‐Time PCR (RT‐qPCR)

Total RNA was extracted from treated HT‐3 cells using TRIzol reagent (Invitrogen, 15596026). One microgram of RNA was reverse‐transcribed into cDNA using a PrimeScript RT reagent kit (Takara, RR037A). Quantitative PCR was performed on a QuantStudio 6 Pro system (Applied Biosystems) using SYBR Green Premix (Takara, RR420A). The thermal cycling protocol consisted of an initial denaturation at 95°C for 30 s, followed by 40 cycles of 95°C for 5 s and 60°C for 34 s. Gene expression was calculated using the 2^−ΔΔCt^ method, with *GAPDH* serving as the endogenous control. The primer sequences used were *SOD2* forward: 5′‐GCTCCGGTTTTGGGGTATCTG‐3′, reverse: 5′‐GCGTTGATGTGAGGTTCCAG‐3′; *DRP1* forward: 5′‐CTGCCTCAAATCGTCGTAGTG‐3‘, reverse: 5′‐GAGGTCTCCGGGTGACAATTC‐3′; *MFN1* forward: 5′‐GAGGTGCTATCTCGGAGACAC‐3‘, reverse: 5′‐GCCAATCCCACTAGGGAGAAC‐3′; *MFN2* forward: 5′‐CTCTCGATGCAACTCTATCGTC‐3‘, reverse: 5′‐TCCTGTACGTGTCTTCAAGGAA‐3′; *GAPDH* forward: 5′‐ACAACTTTGGTATCGTGGAAGG‐3′, reverse: 5′‐GCCATCACGCCACAGTTTC‐3′.

### 2.5 Co‐Immunoprecipitation and Immunoblotting

The cells were collected and lysed in RIPA buffer with protease and phosphatase inhibitors. The technique called for a Mitochondria Isolation Kit (Thermo Scientific, 89874) for mitochondrial fractionation. A BCA test assessed protein concentration. Equal protein quantities (20–30 µg) were separated using SDS‐PAGE and transferred to PVDF membranes. Membranes were probed overnight at 4°C with primary antibodies after blocking with 5% non‐fat milk. For immunoblotting studies, the following primary antibodies—all diluted in TBST with 5% BSA—were used: Cell Signaling Technology (#4494) anti‐phospho‐Drp1 (Ser616) at 1:1000; Cell Signaling Technology (#8570) anti‐Drp1 at 1:1000; Abcam (#ab68155) anti‐SOD2 at 1:2000; Cell Signaling Technology (#77055) anti‐CDK1 at 1:1000; Cell Signaling Technology (#4138) anti‐Cyclin B1 at 1:1000; Cell Signaling Technology (#4661) anti‐VDAC at 1:2000; and Sigma‐Aldrich (#A1978) anti‐β‐actin at 1:5000. Protein bands were visualized using an ECL substrate (Millipore) and photographed using a ChemiDoc system (Bio‐Rad) after being incubated with the appropriate HRP‐conjugated secondary antibodies. ImageJ software was used for densitometric analysis. For co‐immunoprecipitation, 500 µg of whole‐cell lysate was treated with either normal rabbit IgG or 2 µg of anti‐CDK1 antibody (CST, 77055) for a whole night at 4°C. Protein A/G Plus Agarose beads (Santa Cruz, sc‐2003) were then incubated for 2 h. For the input control, 20 µg of whole‐cell lysate was loaded directly for immunoblotting alongside the immunoprecipitated samples.

### 2.6 Mitochondrial Parameter Measurement

MitoSOX Green (Invitrogen) was used to measure mitochondrial superoxide production. Following treatment, cells were exposed to 5 µM MitoSOX reagent in serum‐free medium for 30 min at 37°C. After that, the cells were rinsed, trypsinized, and put back into PBS suspension. Using flow cytometry (BD FACSCelesta), the fluorescence intensity was determined right away and normalized to the protein concentration of the respective samples. An ATP Determination Kit (Invitrogen, A22066) based on a luciferase response was used to measure cellular ATP levels. Luminescence was measured when treated cells were lysed. Using known ATP concentrations, a standard curve was created, and data were reported as nanomoles of ATP per milligram of protein (nmol/mg protein). Citrate synthase and cytochrome c oxidase (Complex IV) enzymatic activity were measured using particular kits (Abcam, ab109911, and ab239712, respectively) in accordance with the manufacturer's instructions. Activities are expressed as nanomoles of substrate converted per minute per milligram of protein and were computed using the linear slope of absorbance change over time.

### 2.7 Mitochondrial Morphology Assessment

To observe mitochondrial networks, cells on glass coverslips were stained with 100 nM MitoTracker Red CMXRos (Invitrogen, M7512) in full medium for 30 min at 37°C. Zeiss LSM 880 microscopes with 63× oil immersion objectives captured confocal images. For quantification, at least 30 cells per condition from three separate experiments were randomly chosen. The mitochondrial network in each cell was classified into one of three categories: predominantly tubular (elongated, > 80% of mitochondria in filamentous form), intermediate (mixed tubular and punctate populations), or fragmented (> 80% punctate, with average particle length < 2 µM). The percentage of cells in each category was calculated. The ImageJ MiNA macro tool determined the average mitochondrial particle length per cell.

### 2.8 Lentiviral Knockdown of CDK1

Lentiviral particles with a short hairpin RNA (shRNA) targeting human CDK1 (TRCN0000000037, Sigma‐Aldrich) or a non‐targeting control shRNA (SHC002) were employed. HT‐3 cells were infected with viral particles in the presence of 8 µg/ml Polybrene. Stable pools were chosen 72 h after transduction and treated with 2 µg/ml puromycin for a week. Western blot analysis was used to validate the knockdown effectiveness prior to experimentation.

### 2.9 Immunohistochemistry

Deparaffinized and rehydrated 4 µM thick formalin‐fixed, paraffin‐embedded tissue slices. Sections were heated in citrate buffer (pH 6.0) to retrieve the antigen. 3% H_2_O_2_ inhibited endogenous peroxidase activity. Sections were incubated overnight at 4°C with primary antibodies against p‐Drp1 (Ser616) or total Drp1 after blocking with normal goat serum. A biotinylated secondary antibody and streptavidin‐HRP combination were used, followed by DAB development and hematoxylin counterstaining. Two blinded pathologists assessed staining intensity. A histoscore (H‐score) was generated by multiplying the intensity score (0–3) by the positive cell percentage.

### 2.10 Assay for Apoptosis

Apoptosis was assessed by flow cytometry and Annexin V‐FITC/PI labeling (BD Pharmingen, 556547). Following the indicated treatment, HT‐3 cells were collected, washed with cold PBS, and stained in accordance with the manufacturer's instructions. BD FACSCelesta was used to analyze the samples, and the early and late apoptotic/necrotic fractions (Annexin V^+^/PI^+^) were added to determine the total number of apoptotic cells.

### 2.11 Statistical Analysis

Three biological replicates were required for all studies. Data are shown as mean ± SEM. When necessary, unpaired or paired Student's t‐tests compared two groups. One‐way or two‐way analysis of variance (ANOVA) was used for multiple group comparisons, followed by Tukey's or Dunnett's post‐hoc test. For categorical mitochondrial morphology data, a chi‐square test was applied. Statistics were deemed significant at *p* < 0.05. All analyses were conducted using GraphPad Prism 9.0.

## 3 Results

### 3.1 Phosphorylated Drp1 is Diminished in Human Cervical Carcinoma Specimens

**Figure 1:**
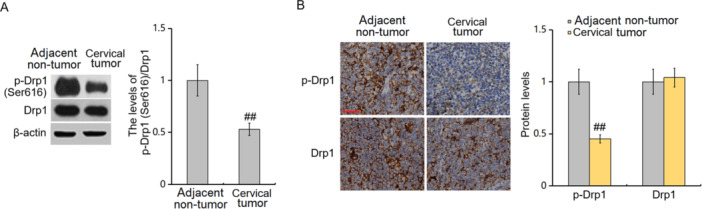
Analysis of phosphorylated Drp1 (Ser616) in cervical cancer tissues. (A) Representative western blot and quantitative analysis of p‐Drp1 (Ser616) and total Drp1 protein levels in adjacent non‐tumor (N) and cervical cancer (T) tissues (*n* = 6 pairs). Data show the p‐Drp1/Drp1 ratio. (B) Representative immunohistochemistry images and quantification of p‐Drp1 and Drp1 staining in tissue sections (n = 8 pairs). Staining intensity was scored as a histoscore. Scale bar, 50 μM. Data are mean ± SEM. ##*p* < 0.01 versus non‐tumor tissue (paired *t*‐test).

We first sought to determine the status of Drp1 activation, a pivotal regulator of mitochondrial fission, in clinical cervical cancer. Western blot analysis of paired tissue samples revealed that the ratio of phospho‐Drp1 (Ser616) to total Drp1 was significantly lower in cervical cancer tissues compared to adjacent non‐tumor mucosa (Figure 1A). This reduction in the active form of Drp1 was corroborated by immunohistochemical staining, which demonstrated substantially weaker p‐Drp1 immunoreactivity in malignant epithelial cells, while total Drp1 protein levels remained comparable between groups (Figure 1B). The histoscore analysis of 8 paired samples confirmed this pattern. The consistent decrease in p‐Drp1 across tumor samples indicates that suppressed mitochondrial fission may be a characteristic of cervical cancer pathogenesis.

### 3.2 β‐Elemene Exerts Dose‐Dependent Cytotoxic Effects on Cervical Cancer Cells

**Figure 2:**
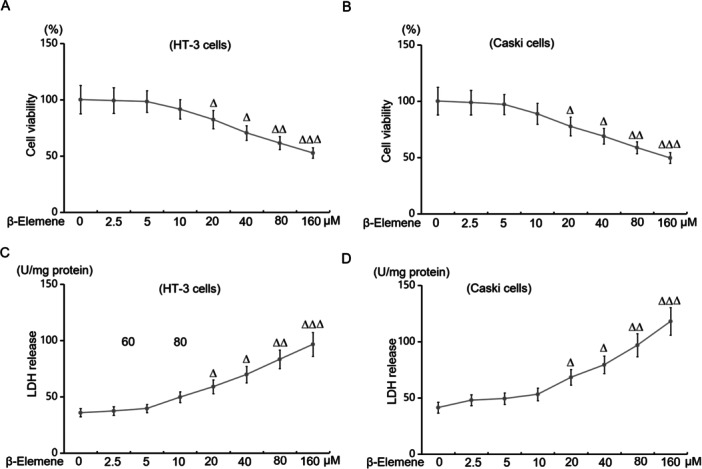
Cytotoxic effects of β‐elemene on cervical cancer cells. HT‐3 and Caski cells were treated with indicated concentrations of β‐elemene (0–80 µM) for 48 h. (A, B) Cell viability measured by CCK‐8 assay, expressed as a percentage of vehicle control. (C, D) Lactate dehydrogenase (LDH) release was assayed in culture supernatant, normalized to total cellular protein (U/mg protein). Data are mean ± SEM from three independent experiments. ^Δ^ *p* < 0.05, ^ΔΔ^ *p* < 0.01, ^ΔΔΔ^ *p* < 0.001 versus 0 µM group (one‐way ANOVA).

To evaluate the anti‐proliferative potential of β‐elemene, we treated HT‐3 and Caski cervical cancer cells with increasing concentrations of the compound for 48 h. Cell viability, assessed by CCK‐8 assay, declined in a concentration‐dependent manner in both cell lines (Figure 2A,B). Concomitantly, measurement of LDH release into the culture medium demonstrated a progressive increase in cytotoxicity, confirming that loss of cell viability was associated with plasma membrane damage (Figure 2C,D). Similarly, β‐elemene reduced viability and raised LDH release in HeLa cells, as shown in Figure S1. These data establish that β‐elemene effectively inhibits the growth and integrity of cervical cancer cells across multiple cell lines of distinct HPV status.

### 3.3 Treatment With β‐Elemene Causes Mitochondrial Oxidative Stress and Functional Impairment

**Figure 3:**
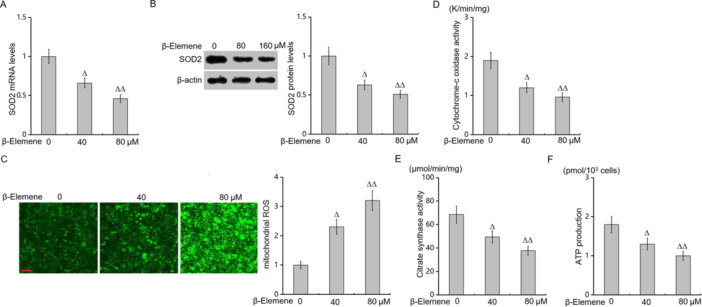
β‐elemene induces mitochondrial oxidative stress and functional impairment in HT‐3 cells. Cells were treated with 40 or 80 µM β‐elemene for 48 h. (A) *SOD2* mRNA levels were analyzed by RT‐qPCR and normalized to *GAPDH*. (B) SOD2 protein levels assessed by western blot, quantified relative to β‐actin. (C) Mitochondrial superoxide was measured using MitoSOX Green; fluorescence was quantified. Scale bar, 200 μm. (D) Cytochrome c oxidase (Complex IV) activity. (E) Citrate synthase activity. (F) Cellular ATP levels. Enzyme activities are nmol/min/mg protein. Data are fold‐change relative to control, mean ± SEM. ^Δ^ *p* < 0.05, ^ΔΔ^ *p* < 0.01 versus 0 µM group (one‐way ANOVA).

Given its cytotoxic effects, we tested whether β‐elemene alters mitochondrial redox homeostasis in HT‐3 cells. Treatment with 40 or 80 µM β‐elemene dramatically reduced the expression of the mitochondrial antioxidant enzyme SOD2 at both transcriptional and translational levels, as shown by quantitative PCR and western blotting (Figure 3A,B). This inhibition of cellular defense was coupled with a significant increase in mitochondrial superoxide, as assessed by MitoSOX Green fluorescence (Figure 3C). Next, we examined the functional consequences of this oxidative insult. The enzymatic activity of cytochrome c oxidase, a critical component of the electron transport chain, was substantially reduced (Figure 3D). Citrate synthase activity, a marker of mitochondrial matrix integrity and abundance, was also significantly attenuated (Figure 3E). Consistent with impaired respiratory chain function, cellular ATP content was depleted in a dose‐dependent fashion (Figure 3F). As a result, the compound causes a significant increase in oxidative stress inside target cells' mitochondria and a broad failure of mitochondrial bioenergetic capacity.

### 3.4 Mitochondrial Network Morphology Undergoes Extensive Fragmentation

**Figure 4:**
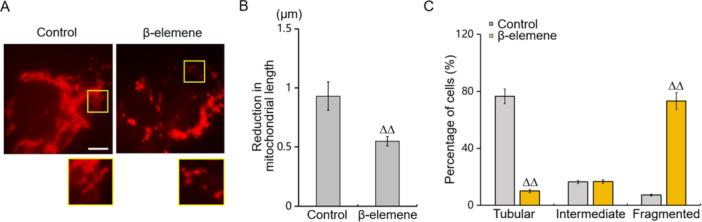
β‐elemene triggers mitochondrial fragmentation in HT‐3 cells. Cells were treated with 80 µM β‐elemene for 48 h. Mitochondria were stained with MitoTracker Red CMXRos and imaged by confocal microscopy. (A) Representative fluorescence images. Scale bar, 10 µM. (B) Quantification of average mitochondrial length from ≥ 30 cells per condition. Data are mean ± SEM. ^ΔΔ^ *p* < 0.01 versus control (unpaired two‐tailed Student's *t*‐test). (C) Morphology classification of mitochondria into tubular, intermediate, or fragmented categories. Percentages of cells in each category are shown. ^ΔΔ^ *p* < 0.01 versus control (chi‐square test).

To determine if functional deficits were linked to structural alterations, we visualized mitochondrial morphology using MitoTracker Red. Confocal microscopy of control cells revealed an elongated, interconnected tubular network (Figure 4A). In stark contrast, treatment with 80 µM β‐elemene for 48 h caused severe fragmentation of mitochondria into punctate, spherical structures (Figure 4A). Quantitative analysis confirmed a significant reduction in the average length of mitochondrial units (Figure 4B). Categorical quantification showed that 76.7% ± 5.2% of control cells displayed predominantly tubular networks, while after β‐elemene treatment, 73.3% ± 5.8% of cells exhibited fragmented mitochondria (Figure 4C). The compound thus precipitates a profound shift in mitochondrial dynamics towards fission.

### 3.5 Drp1 Activation and Translocation Mediate the Fission Process

**Figure 5:**
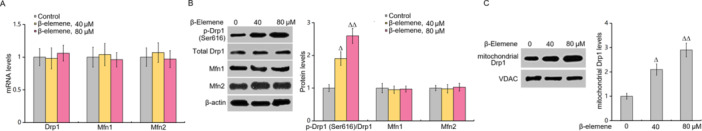
β‐elemene activates Drp1 phosphorylation and mitochondrial translocation. HT‐3 cells were treated with 40 or 80 µM β‐elemene for 48 h. (A) mRNA levels of *DRP1*, *MFN1*, and *MFN2* by RT‐qPCR. (B) Western blot and quantification of p‐Drp1 (Ser616), total Drp1, Mfn1, and Mfn2 in whole‐cell lysates. (C) Drp1 levels in mitochondrial fractions, normalized to VDAC. Data are fold‐change relative to control, mean ± SEM (n = 3). ^Δ^ *p* < 0.05, ^ΔΔ^ *p* < 0.01 versus 0 µM group (one‐way ANOVA).

We probed the molecular mechanism underlying the observed fragmentation. mRNA levels of fission regulator DRP1 and fusion mediators MFN1 and MFN2 were unaltered by β‐elemene treatment, excluding transcriptional control (Figure 5A). However, western blot analysis of whole‐cell lysates revealed a specific and dose‐dependent increase in Drp1 phosphorylation at the activating Ser616 site, without changes in total Drp1, Mfn1, or Mfn2 protein levels (Figure 5B). Furthermore, analysis of purified mitochondrial fractions demonstrated a concomitant enrichment of Drp1 protein within mitochondria (Figure 5C). β‐Elemene specifically promotes the post‐translational activation and organellar recruitment of Drp1 to execute fission.

### 3.6 The CDK1/cyclin B1 Complex Is Engaged by Β‐Elemene

**Figure 6:**
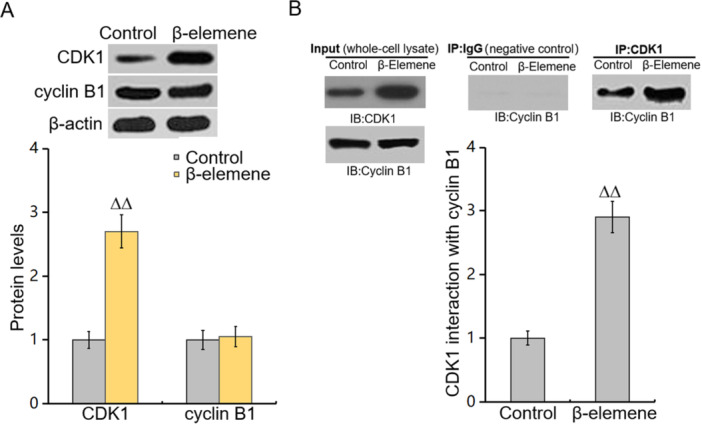
β‐elemene enhances the CDK1‐cyclin B1 interaction. HT‐3 cells were treated with 80 µM β‐elemene for 48 h. (A) Western blot and quantification of CDK1 and cyclin B1 protein levels. (B) Co‐immunoprecipitation analysis. Input lysates and IP fractions were immunoblotted as indicated. Lysates were immunoprecipitated with anti‐CDK1 or control IgG, then immunoblotted for cyclin B1. The graph shows cyclin B1 co‐precipitated with CDK1, normalized to input. Data are mean ± SEM (*n* = 3). ^ΔΔ^ *p* < 0.01 versus 0 µM group (unpaired *t*‐test).

To identify upstream signaling events leading to Drp1 activation, we analyzed the mitotic kinase CDK1 and its regulatory partner cyclin B1. While total cyclin B1 protein was unchanged, CDK1 levels were modestly increased in HT‐3 cells treated with β‐elemene (Figure 6A). More importantly, co‐immunoprecipitation assays demonstrated a robust enhancement of the physical interaction between CDK1 and cyclin B1 in response to treatment (Figure 6B). Input controls confirmed equal loading, and the co‐precipitated cyclin B1 signal was normalized to input. This indicates that β‐elemene facilitates the formation of the active CDK1/cyclin B1 kinase complex.

### 3.7 Genetic Ablation of CDK1 Blocks β‐Elemene‐Induced Mitochondrial Abnormalities and Apoptosis

**Figure 7:**
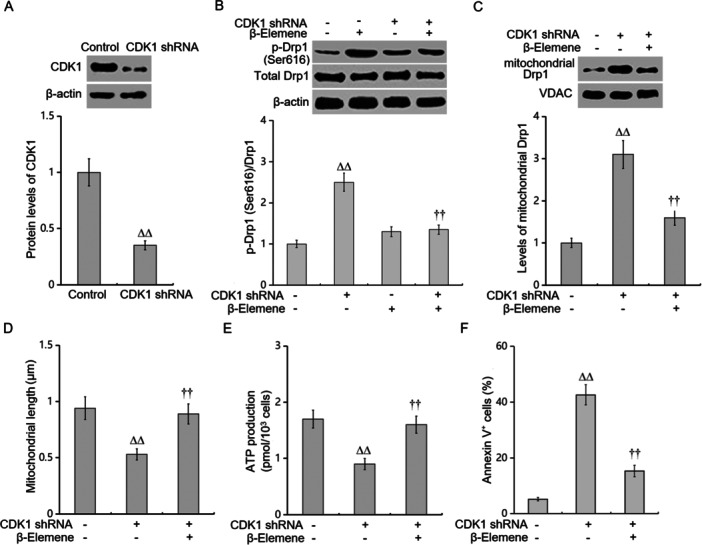
CDK1 knockdown rescues β‐elemene‐induced mitochondrial defects. Cells transduced with control (shCtrl) or CDK1‐targeting (shCDK1) shRNA were treated with 80 µM β‐elemene for 48 h. (A) Western blot confirming CDK1 knockdown. (B) Western blot and quantification of p‐Drp1 (Ser616) and total Drp1 in whole‐cell lysates from shCtrl and shCDK1 HT‐3 cells treated with vehicle or 80 µM β‐elemene. (C) Analysis of mitochondrial Drp1 levels. (D) Quantification of average mitochondrial length. (E) Measurement of cellular ATP levels. (F) Percentage of Annexin V+ cells (total apoptotic cells). Data are mean ± SEM (*n* = 3). ΔΔ*p* < 0.01 versus shCtrl vehicle; ††*p* < 0.01 versus shCtrl + β‐elemene (two‐way ANOVA with Tukey's post‐hoc test).

To demonstrate a causal function for CDK1, we used lentiviral shRNA to knock down its expression in HT‐3 cells. Western blot analysis confirmed the successful knockdown (Figure 7A). β‐elemene treatment of control shRNA cells resulted in Drp1 phosphorylation and mitochondrial translocation, as predicted (Figure 7B,C). These effects were eliminated in CDK1‐knockdown cells. Notably, untreated CDK1‐silenced cells displayed a modestly elevated basal p‐Drp1 signal compared to untreated shCtrl cells, which likely reflects compensatory activation of alternative Ser616 kinases such as ERK or CaMKII [17]. However, β‐elemene failed to stimulate additional significant phosphorylation in the absence of CDK1, confirming that CDK1 is the principal kinase mediating the compound's effect on Drp1. As a result, β‐elemene did not cause substantial mitochondrial fragmentation, and mitochondrial length remained close to control values (Figure 7D). The functional deficiency in ATP generation was likewise dramatically repaired after CDK1 silencing (Figure 7E). Furthermore, the increase in apoptotic cells caused by β‐elemene was inhibited in CDK1‐depleted cells (Figure 7F). In this context, CDK1 activity is crucial for maintaining mitochondrial structure and function.

## 4 Discussion

This study reveals a previously unrecognized molecular pathway through which β‐elemene exerts its anti‐tumor effects in cervical cancer. We demonstrate that β‐elemene induces extensive mitochondrial fragmentation and bioenergetic collapse by activating a specific signaling axis involving CDK1/cyclin B1 and Drp1. The clinical observation of reduced phosphorylated Drp1 in tumor tissues provides a compelling rationale for targeting this pathway. Furthermore, genetic ablation of CDK1 completely rescues the mitochondrial and cytotoxic effects of β‐elemene, establishing a direct causal link. These findings not only elucidate a novel mechanism of action for β‐elemene but also position the CDK1‐Drp1 signaling node as a potential therapeutic vulnerability in cervical cancer.

Our discovery that β‐elemene triggers extensive mitochondrial fission aligns with the broader concept that disrupting mitochondrial dynamics can be an effective anti‐cancer strategy [19]. Numerous cancers exhibit a reliance on balanced mitochondrial dynamics for survival, and tipping this balance toward either excessive fusion or fission can induce cell death [20]. Previous work has shown that pharmacological agents or genetic maneuvers promoting uncontrolled fission often lead to mitochondrial dysfunction and apoptosis [21]. In this context, our results are consistent with the paradigm that pro‐fission stimuli can be cytotoxic to cancer cells. However, the scientific literature also presents a complex picture. Some reports suggest that certain cancer cells depend on elevated fission for proliferation and metastasis [22]. This apparent contradiction may be explained by the concept of “therapeutic window” or differential baseline fission activity. Our initial finding of lower p‐Drp1 levels in cervical cancer tissues suggests a potential baseline state of suppressed fission. This could render cervical cancer cells particularly susceptible to a strong exogenous pro‐fission signal like that generated by β‐elemene, whereas cancers with constitutively high fission might be less affected or even depend on it. This tissue‐specific and context‐dependent role of fission proteins underscores the importance of our clinical observation and suggests that β‐elemene's efficacy might be stratified by the pre‐existing mitochondrial dynamics state of the tumor.

The most significant mechanistic contribution of this work is the identification of CDK1/cyclin B1 as the critical upstream activator of Drp1 in response to β‐elemene. While the anti‐cancer properties of β‐elemene have been documented for various tumors, its molecular targets remain incompletely defined [23]. Previous studies have implicated pathways like apoptosis induction and cell cycle arrest, but a connection to the core mitochondrial fission machinery had not been established [24]. Concurrently, the regulation of Drp1 by various kinases, including CDK1, has been described in other biological contexts, such as during mitosis or neuronal injury [16, 25]. The principal novelty of our study lies in bridging these two separate fields. We provide the first evidence that a natural anti‐cancer compound, β‐elemene, selectively engages this specific kinase complex (CDK1/cyclin B1) to phosphorylate and activate Drp1, thereby initiating a mitochondrial crisis in cervical cancer cells. This is distinct from other known Drp1 kinases, such as Erk or CaMKII, which are activated by different stimuli [26, 27]. Our data showing enhanced CDK1/cyclin B1 interaction without a major change in their total protein levels points to a post‐translational, activation‐centric mechanism rather than simple overexpression. This finding expands the understanding of how extracellular signals can be transduced into profound mitochondrial morphological changes via a cell cycle‐related kinase.

We observed that chronic CDK1 silencing produced a modest elevation in basal p‐Drp1(Ser616). This could arise from compensatory engagement of other kinases, including ERK1/2 or CaMKIIα, that are also capable of phosphorylating Drp1 at this activating residue [17]. Such adaptive baseline phosphorylation has been noted in other contexts where a primary kinase is genetically ablated. Importantly, this compensatory increase did not restore responsiveness to β‐elemene; the compound failed to produce appreciable additional Drp1 activation in CDK1‐depleted cells. Thus, CDK1 functions as the major transducer of β‐elemene signaling to Drp1, whereas basal phosphorylation is governed by redundant, drug‐insensitive pathways.

β‐Elemene treatment led to a cascade of mitochondrial defects: oxidative stress, impaired electron transport chain function, and ATP depletion. These are classic hallmarks of mitochondrial failure and are tightly linked to the induction of cell death [28]. The sequence of events, CDK1 activation leading to Drp1 phosphorylation, followed by mitochondrial translocation, fragmentation, functional decline, and ultimately apoptosis, establishes a clear linear pathway from drug exposure to cellular demise. Annexin V/PI staining confirmed that the observed mitochondrial dysfunction culminates primarily in classical apoptosis. The rescue of every downstream phenotype (Drp1 activation, fragmentation, ATP loss, apoptosis) upon CDK1 knockdown provides exceptionally strong evidence for the centrality of this axis. From a therapeutic perspective, this work suggests that cervical cancers with low basal p‐Drp1 might be primed for this type of intervention. It proposes a novel combination strategy: agents that modulate CDK1 activity or directly force Drp1 activation could be explored in conjunction with existing therapies. Furthermore, monitoring p‐Drp1 levels in tumors could serve as a potential biomarker for predicting sensitivity to β‐elemene or similar pro‐fission agents, moving toward a more personalized treatment approach [29].

Although this study delineates a novel signaling pathway, certain limitations must be acknowledged to guide future research. First, our work was conducted exclusively in cell line models. The physiological relevance of this pathway needs validation *in vivo* using cervical cancer xenograft models to assess tumor growth inhibition and potential side effects. Second, we focused on CDK1 as the critical kinase. Although our co‐immunoprecipitation data show enhanced interaction with cyclin B1, we did not perform an in vitro kinase assay to definitively prove CDK1 directly phosphorylates Drp1 in this context. This remains an important experimental verification. Third, the upstream sensor linking β‐elemene to CDK1/cyclin B1 activation is unknown. β‐Elemene, due to its hydrophobic nature, may integrate into lipid bilayers and perturb membrane‐associated signaling, or induce ROS‐mediated activation of ATM/ATR, both of which can promote CDK1/cyclin B1 assembly [23, 28]. Further studies using chemical proteomics could identify the direct binding partner. How the compound engages the cellular machinery to promote this kinase complex interaction is a key open question. Investigation into upstream receptors or stress sensors would complete the signaling picture. Fourth, although we have now validated the cytotoxic effect in three cell lines with different HPV statuses (HT‐3, Caski, HeLa), future studies employing a wider panel and primary patient‐derived cultures will be essential to confirm generalizability [30]. Although we defined the terminal mode as apoptosis, the detailed apoptotic signaling cascade downstream of mitochondrial fission warrants further exploration.

## 5 Conclusions

In summary, this research identifies a novel cytotoxic mechanism for β‐elemene in cervical cancer. The compound promotes the interaction and activity of the CDK1/cyclin B1 complex, which phosphorylates Drp1. Activated Drp1 translocates to mitochondria, driving excessive fission. This fragmentation causes severe oxidative stress and bioenergetic failure, leading to cell death. The lower levels of active Drp1 found in clinical tumors suggest a therapeutic opportunity. Silencing CDK1 completely blocks this entire cascade, confirming its essential role. These results reveal a new signaling pathway that connects a natural product to mitochondrial dynamics via a cell cycle kinase. This work provides a mechanistic foundation for considering β‐elemene as a potential agent and highlights the CDK1‐Drp1 axis as a target for cervical cancer therapy.

## Author Contributions

**Zhifang Li:** conceptualization, methodology, software, data curation, investigation, validation, writing – original draft, writing – review and editing, resources, project administration. **Ling Tang:** conceptualization, methodology, software, data curation, investigation, validation, project administration, resources, writing – original draft, writing – review and editing. **Mingyan Wang:** investigation, software, formal analysis, validation, project administration, writing – original draft, writing – review and editing. **Qiong Yu:** methodology, investigation, validation, formal analysis, writing – review and editing. **Chen Chen:** investigation, validation, writing – review and editing. **Jiyi Xia:** conceptualization, methodology, software, data curation, supervision, validation, funding acquisition, visualization, project administration, resources, writing – original draft, writing – review and editing. **Xiaolan Yu:** conceptualization, methodology, software, data curation, validation, formal analysis, supervision, resources, project administration, visualization, funding acquisition, writing – original draft, writing – review and editing.

## Supporting information


**Figure S1:** Cytotoxic effects of β‐elemene on HeLa cells. HeLa cells were treated with 0, 20, 40, 60, 80 µM β‐elemene for 48 h. (A) Cell viability was measured by CCK‐8. (B) LDH release. Data are mean ± SEM (n = 3). Δ*P* < 0.05, ΔΔ*P* < 0.01 vs. 0 µM (one‐way ANOVA).

## Data Availability

The data that support the findings of this study are available from the corresponding author upon reasonable request.
